# Influence of Wind Speed on RGB-D Images in Tree Plantations

**DOI:** 10.3390/s17040914

**Published:** 2017-04-21

**Authors:** Dionisio Andújar, José Dorado, José María Bengochea-Guevara, Jesús Conesa-Muñoz, César Fernández-Quintanilla, Ángela Ribeiro

**Affiliations:** 1 Centre for Automation and Robotics, Spanish National Research Council, CSIC-UPM, Argandadel Rey, 28500 Madrid, Spain; jose.bengochea@car.upm-csic.es (J.M.B,-G.); jesus.conesa@csic.es (J.C.-M.); angela.ribeiro@csic.es (Á.R.); 2 Institute of Agricultural Sciences, Spanish National Research Council, CSIC, 28006 Madrid, Spain; jose.dorado@ica.csic.es (J.D.); cesar@ica.csic.es (C.F.-Q.)

**Keywords:** RGB-D images, Kinect sensor limits, depth information, wind speed, woody crops

## Abstract

Weather conditions can affect sensors’ readings when sampling outdoors. Although sensors are usually set up covering a wide range of conditions, their operational range must be established. In recent years, depth cameras have been shown as a promising tool for plant phenotyping and other related uses. However, the use of these devices is still challenged by prevailing field conditions. Although the influence of lighting conditions on the performance of these cameras has already been established, the effect of wind is still unknown. This study establishes the associated errors when modeling some tree characteristics at different wind speeds. A system using a Kinect v2 sensor and a custom software was tested from null wind speed up to 10 m·s^−1^. Two tree species with contrasting architecture, poplars and plums, were used as model plants. The results showed different responses depending on tree species and wind speed. Estimations of Leaf Area (LA) and tree volume were generally more consistent at high wind speeds in plum trees. Poplars were particularly affected by wind speeds higher than 5 m·s^−1^. On the contrary, height measurements were more consistent for poplars than for plum trees. These results show that the use of depth cameras for tree characterization must take into consideration wind conditions in the field. In general, 5 m·s^−1^ (18 km·h^−1^) could be established as a conservative limit for good estimations.

## 1. Introduction

Plant reconstruction by non-destructive methods is of high value for decision making processes [1]. The use of sensors for plant characterization may lead to a better knowledge of the processes involved in plant development all over throughout the life cycle and may improve the decisions taken for plant production, contributing to create new protocols to enhance the profitability of crops [2].

Current techniques for plant characterization vary from manual to fully automatic, using a great variety of imaging to non-imaging technologies [3]. Plant characterization using machine vision by portable imaging and analysis software is the most investigated technique [4]. RGB cameras have been widely used for phenology monitoring [5], plant geometric characterization [6], nitrogen application [7], yield monitoring [8] and weed/crop discrimination [9]. These cameras can acquire images with a high resolution and at a low cost. However, their limited capacity to provide spectral and structural information is a deterrent to their usage in plant reconstruction. In addition, under outdoor conditions, the variable and uncontrolled illumination and the presence of shadows may represent a serious problem [10].

Although various systems can be used to estimate plant parameters, the third dimension is usually missing. Distance sensors, which measure the distance from the sensor to an object, allow one to create 3D models, increasing the effectiveness in plant description [11]. There are two main types of distance sensors available: ultrasonic and light detection and ranging (LiDAR). Both of them can be used for plant height and volume estimation. The ultrasonic sensors measure the reflected echo that the sensor transmits as sound waves of high frequency toward an object. They have been used for tree canopy characterization [12] and for discrimination between plant types (grasses and broad-leaved) [13,14]. Distance measurements may be improved by using a terrestrial laser scanner (TLS). This is a simple device that is able to provide 2D or 3D plant models by displacing the sensor along the row and storing the relative position of the sensor. The sensor measures the distance to the impacted object with a laser beam by time-of-flight method or phase-shift measurement. Llorens et al. [15] used a dual methodology of LiDAR and ultrasonic sensors in the characterization of tree canopies, concluding that LiDAR assessments were the most accurate. Andújar et al. [16] obtained promising results using a TLS to characterize the profile of poplar trees. Various other systems have also been explored for plant characterization in precision agriculture, including radar systems [17], hemispherical photography [18], stereo-vision [19], and magnetic resonance or X-ray visualization [20].

In the last few years, a new geometric reconstruction concept based on the use of depth cameras has arisen [21]. The Kinect device, originally designed by Microsoft to track body position and movement for video-games and digital entertainment, has also been used for plant characterization in agriculture. Paulus et al. [22] compared two low-cost 3D imaging systems (a Kinect device and a David laser scanning system) with a high precision laser scanner to estimate volumetric shape of sugar beet leaves and taproots and of wheat ears. They concluded that the two low-cost sensors could replace the expensive laser scanner in some of the plant phenotyping scenarios. Chéné et al. [23] developed an algorithm to segment depth images of plant obtained from top view. They showed the potential of this sensor for volume estimation, leaf curvature and morphology, or the use for pathogens detection. Similarly, Andújar et al. [24] proposed the use of depth cameras for determining the weed volume and defining herbicide spraying date [25]. Wang and Li [26] used volume estimation from Kinect models for onions, showing that volume and fruit density were directly related with Kinect measurements.

The use of sensors located on field platforms may allow monitoring plant growth under natural environments. However, these sensors may be influenced by different environmental conditions present throughout the growing season. Recent studies have showed the influence of light on sensors measurements [27]. Although daytime light contamination has limited the potential use of Kinect v1 (projected light pattern and triangulation) sensors in open spaces [28], the new version (Kinect v2) uses a completely different measurement principle (time-of-flight) that should overcome, at least partially, this problem [29,30,31]. Other parameters such as wind can modify the captured information related to the plant structure. When a plant is moving due to wind conditions sensor readings can differ along the time. Decision making requires standardized conditions when the information is captured. Since wind speed cannot be controlled outdoors, the influence of its effect should be quantified. The overall objective of this work was to assess the effect of wind speed on measurements recorded by a Kinect v2 sensor related to plant height, volume and leaf area, as well as to establish the limits and errors in these parameters measured in cultivated plants and associated with different wind speeds.

## 2. Materials and Methods

### 2.1. Hardware, Software and Other Equipment

Microsoft Kinect is a RGB-D camera that uses the time-of-flight method for the calculation of the camera distance to an object. The Kinect one is composed by an RGB camera of 1080 p, a depth camera, an infrared (IR) camera and an array of microphones. Kinect v2 has a self-adaptation of the exposure time of the RGB image. Thus, by automatically adapting this parameter the sensor obtains brighter images. However, this limits the number of frames that are captured in a time interval to a minimum frame rate of 15 fps. The time-of-flight system modulates a camera light source with a square wave. It uses phase detection to measure the time it takes light to travel from the light source to the object and back to the sensor, estimating distance from the results. The system calculates distance from the speed of light in air by estimating the received light phase at each pixel with knowledge of the modulation frequency. The RGB camera creates raw color images with a 1920 × 1080 resolution. Kinect only provides an array of pixel RGB values that are converted into a proper Windows Presentation Foundation (WPF) representation. The IR camera can take a clear view into the dark. It has a resolution of 512 × 424 pixels. The sensor allows tracking of IR reflective objects while filtering out IR lights. The Kinect v2 has a wider field of view in the IR being able of acquiring depth information at 70 degrees horizontally and 60 degrees vertically. This effect allows to capture objects closer to the camera and still in its field of view. The camera is effective at distances from 0.5 m to 4.5 m. The use of reconstruction tools allows the creation of bigger models by displacing the sensor and using custom software based on the Iterative Closest Point (ICP) algorithm [32].

The acquisition process was based on an Intel desktop computer with Windows 8 supported by Kinect SDK, which helped to acquire data by classes, functions and structures that also provided options to combining more than one sensor. The SDK provides the necessary drivers for the sensor, and some sample functions that were implemented for the measurements combined with some OpenCV functions [33]. The RGB, depth and infrared images use classes and functions to the camera acquisition process and, if applicable, also coordinate the images acquired by different sensors. The software creates a point cloud by detecting the overlapping areas in sequential frames taken during the acquisition process by assessing the relative position of the sensor for each frame. The overlapped areas allow the creation of the final 3D model and removal of outliers in the mesh.

**Figure 1:**
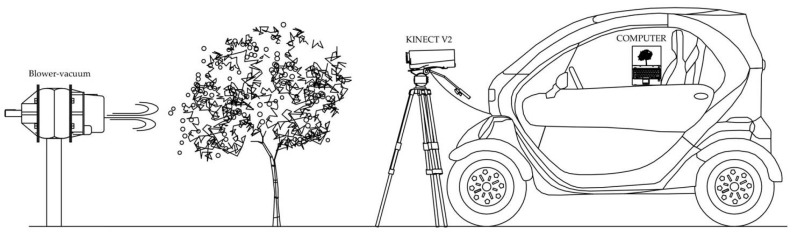
Schematic design of the portable system electrically powered at 220 V by an electric car.

The assessment of the influence of wind speed was conducted by placing the sensor at 1 m height and at 1 m of distance from the tree. The sensor was located on a tripod and turned from 45° to −45°, allowing a full view of the tree. The time to complete the acquisition was 10 s from the top (45°) to the ground view (−45°). The system was supplied with electric power by a field vehicle that allows field measurement and also the storage of the additional devices needed during the process (Figure 1). A set of measurements were conducted at different wind speeds: 0, 2.5, 5, 7.5, and 10 m·s^−1^. Measurements were performed on a sunny cloudless day without natural wind, to avoid the influence of meteorological parameters other than the purpose of this study, i.e., wind speed in a fix direction. In order to create the wind draft a hand-held blower-vacuum (Stihl^TM^ model SH 86, Stihl Inc., Virginia Beach, VA, USA) equipped with a plastic tube was used to artificially create the windy conditions. The wind speed was assessed with a portable anemometer (Testo model 410-1, Testo, Lenzkirch, Germany) at each measurement with a precision of ±0.2 m·s^−1^. Consequently, under these artificial but controlled conditions we assessed the isolate effect of wind on the 3D models created with a RGB-D camera.

### 2.2. Site Study and Field Measurements

Field experiments were conducted on tree plantations located at the experimental farm “La Poveda” (Arganda del Rey, Madrid, Central Spain) during May 2016 and April 2017. Measurements were taken at direct sunlight in a sunny day (approx. 40,000 lux) and without any shading structure. Two tree species with contrasting shape and plant structure were considered: poplar (*Populus* spp.) and plum (*Prunus domestica* L.).These two species were selected for their agronomic importance in the study area, which were naturally available on the experimental farm. The first study was conducted on poplar grown as short rotation coppice, with 1-year old trees, scanning a total of fourteen trees at different wind speeds. The distance between trees was 0.5 m. Trees were selected randomly within the field, but including a representative sample of the population (within 95% confidence interval of mean plant height; data not shown). The adjacent trees were separated with solid wooden sheets in order to avoid interferences from neighboring tree leaves in the model. At sampling time, poplar heights ranged from 0.4 m to 0.6 m. A second study was conducted on a 2-year old plum orchard following the same procedures described previously. Fourteen trees were randomly selected covering the variability in height present within the field.

**Figure 2:**
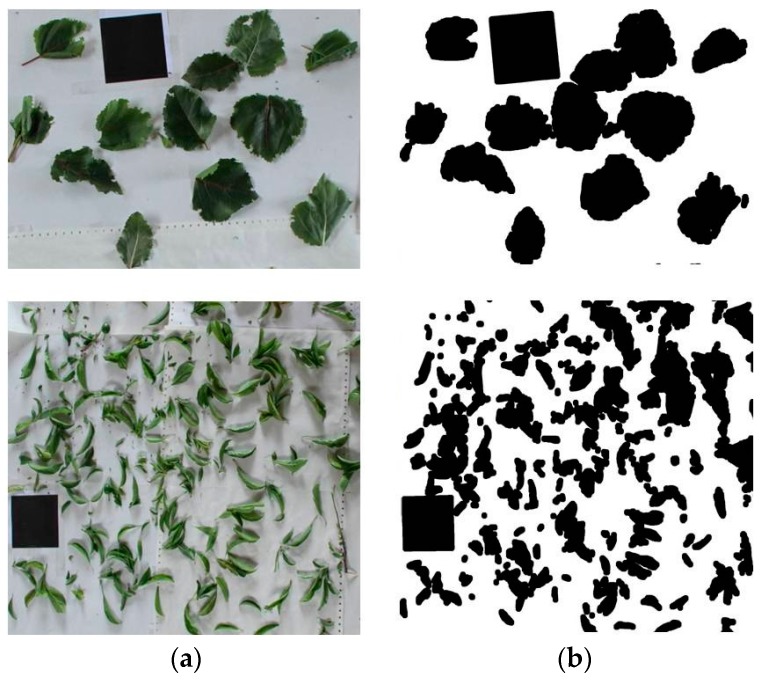
RGB images (**a**) used to quantify the leaf area, after their transformation to binary images (**b**) and subsequent application of the Otsu’s thresholding method. Upper side corresponds to a poplar sample. Down side corresponds to a plum-tree sample. A 100 cm^2^ black square was included in each image as reference area.

After the Kinect measurements, the actual height of every tree was manually measured. Thereafter, trees were cut for determination biomass and Leaf Area (LA). Biomass values were determined by drying the samples (stems plus leaves) of the ten trees in an oven at 78 °C during 48 h and weighting the dry biomass. The LA was calculated from the set of images by placing all the leaves on a white surface. As a reference surface, a standard 100 cm^2^ black square was also placed in the image in order to calculate, by correlation, the leaf area of each sample (Figure 2). The images were obtained with a Canon EOS 7D (Canon, Tokyo, Japan) camera fitted with a 50 mm lens. The RGB images were transformed to binary images. A linear combination of the RGB planes with coefficients (r = − 0.884, g = 1.262, b = − 0.311) was performed. The applied coefficients were obtained by a genetic algorithm optimization process [34] that proved to perform better than Excess Green coefficients (ExG = 2G-R-B) [35]. In the resulting grey level image, the green objects (plants) appear bright, in contrast to objects with a different color which appear dark. Then, the Otsu’s thresholding method [36] was applied to separate the objects pixel-wise into foreground (plants) and background in a binary image. From this image, the values marked as black denoted leaf area and those pixels off the range, which corresponded to white, denoted the background.

### 2.3. Data Processing

**Figure 3:**
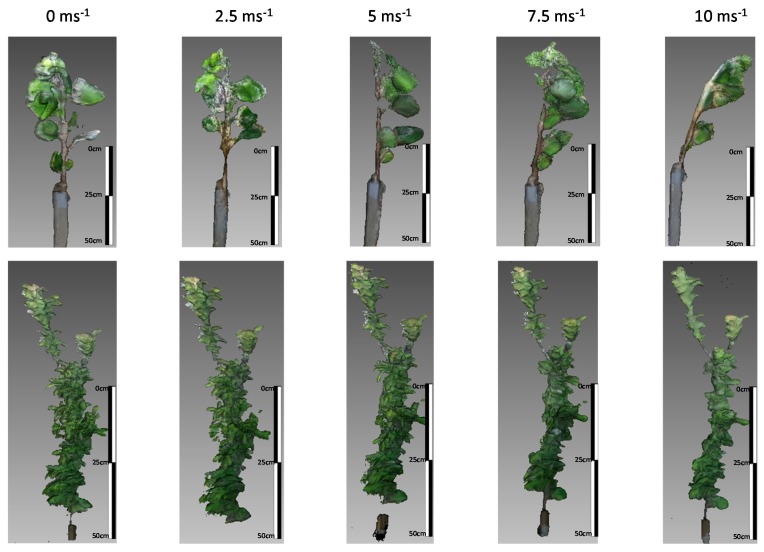
Example of poplar (figures on top) and plum (figures at the bottom) tree models created at different wind speeds, from 0 to 10 m·s^−1^.

The raw data were recorded using Kinect Studio in video mode during a whole period including every wind speed. Thereafter, the file was divided in sections of time corresponding to each wind speed. The meshes were then reconstructed using Kinect fusion algorithm (Microsoft, Redmond, WA, USA). The parameters selected corresponded to Volume Max Integration Weight = 300, Volume Voxels per meter = 128 and Volume Voxels Resolution = 384. From the processed mesh, the desired parameters were calculated by offline processing using the open software Meshlab^®^ (University of Pisa, Italy) (Figure 3). The processing tools incorporated into the software use filters to remove duplicated points, unreferenced vertices, and null faces. The software allows the cleaning, smoothing, visualization and processing of the acquired data readings and plots them as new meshes. The point cloud was processed in two steps [21,37].

(a) Data outliers and noise in the point cloud were filtered out. The filter identifies the points with no connection (1 cm out of the grid and removes them. (b) Tree volumes were calculated from the mesh by computation of polyhedral Mass properties [37]. The algorithm locates the body’s center of mass and computes the moment of inertia about various axes in a dynamic simulation in rigid bodies considering a uniform density. The algorithm is based on a reduction of the volume integrals, minimizing the errors that result from poorly conditioned alignment of polyhedral faces. The volume integrals of a polyhedron are simultaneously computed in a single walk over the boundary of the polyhedron. The algorithm computed the normal, weighting the product over the k-nearest neighbors. The obtained isosurface is approximated from the normal field, whose gradient is related to the indicator function that describes the isosurface. Then, the mesh volume was obtained.

Information extracted from models was analyzed and compared with ground truth data. From the model, parameters regarding plant volume, maximum height and surface were extracted. These values were correlated with actual parameters of height, dry biomass and LA. Pearson’s correlation coefficients were used in the evaluation on simple linear relationships between the data from models and ground truth data. The analyses were repeated for both kinds of trees. The correlation analysis provided the initial information for the scatter plots comparing the cited parameters. The scatter diagrams provided the functional relationship existing between actual parameter and the models at different wind speeds.

## 3. Results and Discussion

**Figure 4:**
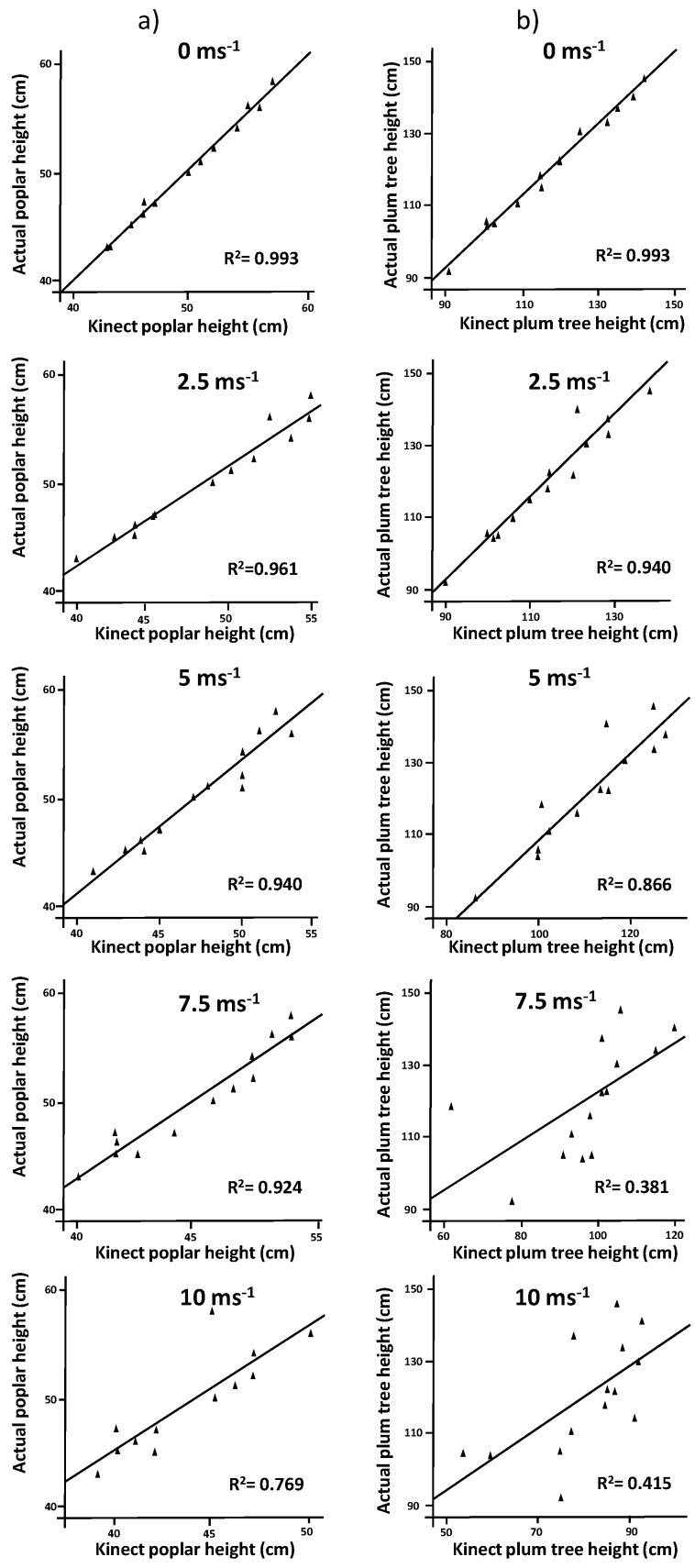
Regression analysis comparing actual height vs. model height for (**a**) poplar trees and (**b**) plum trees at wind speeds ranging from 0 to 10 m·s^−1^.

**Figure 5:**
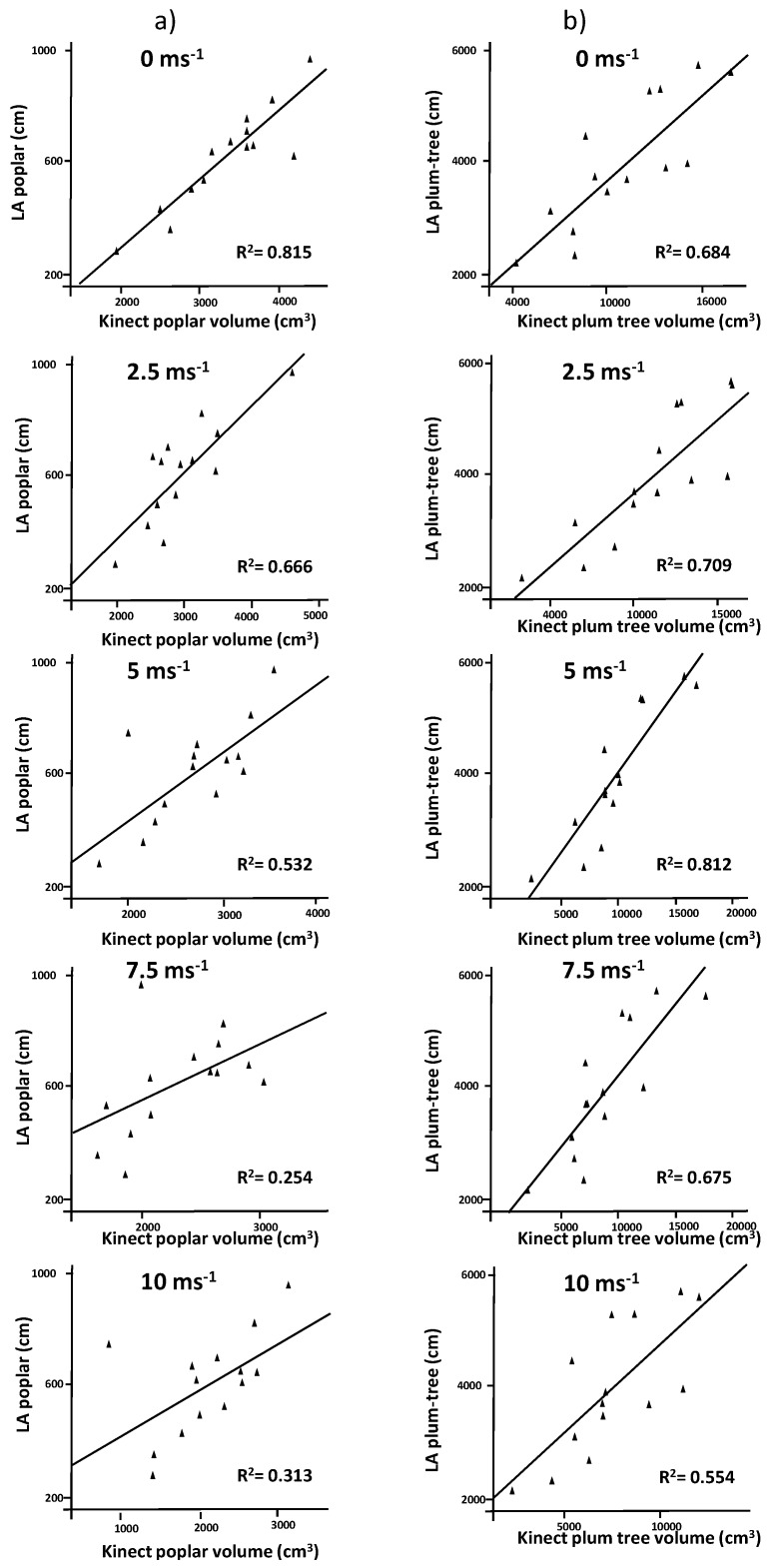
Regression analysis comparing Leaf Area (LA) vs. tree volume for (**a**) poplar trees and (**b**) plum trees at wind speeds ranging from 0 to 10 m·s^−1^.

**Figure 6:**
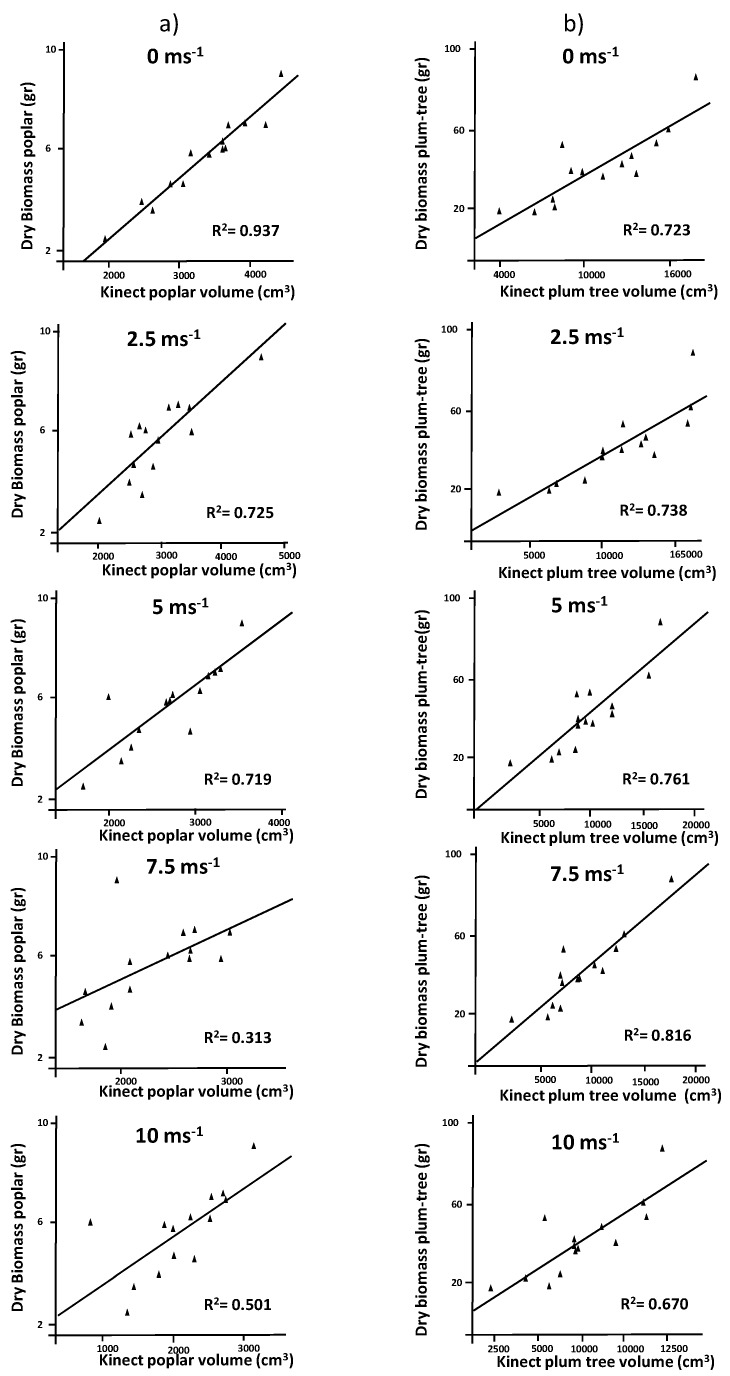
Regression analysis comparing dry biomass (g) vs. tree volume for (**a**) poplar trees and (**b**) plum trees at wind speeds ranging from 0 to 10 m·s^−1^.

In general, variability in Kinect measurements, noise and invalid pixels increased as wind speed increased (Figure 4, Figure 5 and Figure 6). This was expected since the wind moved the trees during data acquisition, changing their position. At null and low wind speeds, the actual height, LA, and biomass showed good correlations with those estimated from the models. However, correlations decreased as wind speed increased.

Actual poplar height ranged from 43 to 58 cm (average: 50.6 cm). These values were strongly correlated (r = 0.996) with those measured by the Kinect v2 under no wind conditions. When wind speed increased to 2.5, 5 and 7.5 m·s^−1^, correlations decreased slightly (r = 0.980, r = 0.970 and r = 0.961, respectively). In contrast, the mean absolute error (MAE) increased from 0.21 in the absence of wind to 1.21 for 2.5 and 5 m·s^−1^, and to 1.14 for 7.5 m·s^−1^. Similarly, the root mean square errors (RMSEs) increased with the wind speed (2.21, 2.14, 2.00 and 5.50 cm for 2.5, 5, 7.5 and 10 m·s^−1^, respectively) relative to the windless scenario (0.21 cm). Nevertheless, actual and estimated measurements of poplar height were still significantly correlated (Figure 4a). In general, although Kinect measurements tended to slightly underestimate plant height when wind speed increased in a fixed direction, we can conclude that this parameter could be satisfactorily estimated by this method up to 7.5 m·s^−1^.

In the case of plum trees, a good agreement was observed between actual and model estimated tree height up to 5 m·s^−1^ wind speed. Actual tree height ranged from 91 to 142 cm (average: 118 cm). The Kinect v2 measurements achieved correlations of r = 0.996, r = 0.970 and r = 0.930 for wind speeds of 0, 2.5 and 5 m·s^−1^, respectively. No significant correlations were obtained with higher wind speeds. MAE resulted in values of 2.14, 3.71 and 4.21 cm in the absence of wind and for the two lower wind speeds. When speeds increased to 7.5 and 10 m·s^−1^, MAE increased up to 12.21 and 17.07 cm. Regression analysis showed good fittings till wind speeds of 5 m·s^−1^ (Figure 4b). Apparently, plum trees were more affected by high wind speeds than poplar trees. The model showed good estimations until 5 m·s^−1^. In general, plum-trees compared with poplar trees were easier affected by higher wind speeds.

The calculation of maximum height using Kinect v2 sensors may have various sources of errors. Wind moves the top of the trunk and the small leaves located in that area affecting the reconstruction of this parameter. This measurement could be improved by analyzing data without filters. However, under these conditions, noise and invalid pixels increase and volume calculation led to worse predictions. The errors associated with the Kinect sensor have been assessed for the previous [38] and the new version [39]. The first version of Kinect was unable to detect branches smaller than 6 mm width. The results were tested for distances of 0.5 to 1.25 m with similar results. In the case of Kinect v2, the range of measurement is higher and error tended to decrease. Under field and laboratory conditions and no influence of other external factors, absolute errors lower than 12 mm were obtained when cotton plants were measured [39]. The accuracy of the system depends not only on the sensor; the target object highly influences the accuracy of measurements. Azzary et al. [21] compared manual and Kinect v1 measurements of plant basal diameter and plant height, concluding that the underestimation or overestimation depends on the plant species and the characteristic of the plant to measure. In our study, models for large leaves were more accurate than those with small leaves. In general, solid objects create more accurate models than complex shapes such as trees. In a study conducted to estimate the dimensions of commercial onions, a 2 mm error in diameter was obtained [26]. The study compared the models obtained with Kinect v1 sensor and RGB cameras. Although both sensors underestimated onion dimensions, depth images created more accurate models than those obtained with RGB images. In a similar study, Andújar et al. [40] showed an error higher than 1 cm when cauliflower dimensions were measured with the first version of Kinect. Those errors could be reduced with Kinect v2, which have showed lower error. However they depend of several parameters such as object reflection, distance object-sensor, foreground, etc. [41]. Measuring the shapes of trees and other plants is an important challenge due to their complex shape.

The parameters extracted from 3D models allowed to characterize leaf area, photosynthetic active area, leaf orientation, dynamics of plant growth, etc. These parameters are directly related with phenotyping processes.

In poplar trees, LA was highly correlated with volume estimation in models created at 0 and 2.5 m·s^−1^ and correlation was still significant at 95% at 5 m·s^−1^. Higher wind speed resulted in not significant correlations. The regressions between poplar volume and LA obtained from the RGB images indicate good agreements at wind speeds below 2.5 m·s^−1^ (Figure 5a). Above this speed wind impedes a good estimation of LA. Since, LA is a parameter directly related with plant growth and productivity and it is a good indicator for nutrient application, the use of sensors in precision applications in poplars should be avoided when wind conditions exceed 2.5 m·s^−1^. In plum trees, model measurements improved significantly compared to poplar trees. Correlations were significant under all wind conditions. However, regression results suggest a decrease in the accuracy when wind speed exceeds 5 m·s^−1^ (Figure 5b). The differences in shape between poplar and plum trees created different patterns in the models. Plum trees were more elongated and internal parts of the trees were less occluded, improving LA estimation under windy conditions.

The actual biomass and the volume estimated using the Kinect v2 were consistent at different wind speeds for the two tree species studied (Figure 6). Poplar trees showed correlations of r = 0.968, r = 0.851 and r = 0.848 for wind speeds of 0, 2.5 and 5 m·s^−1^, respectively. Regression analysis showed similar results. Tree biomass was not properly estimated when wind speeds exceeded 5 m·s^−1^ (Figure 6a). In plum trees, the relationship between actual biomass and the estimated volume was significant at 99% at all wind speeds. However, dispersion of values was greater at high wind speeds (Figure 6b). Volume quantification using Kinect sensors has been tested previously [32]. These studies concluded that plant volume can be properly estimated although with some errors. However, when working with solid objects, such as onions or cauliflowers, estimated values were more similar to real objects [26,40]. When working with whole plants, many points of the cloud impact on internal parts, introducing some errors [2]. In order to reduce these errors, algorithms more accurate than those used for characterization of solid plant products are required.

Structural parameters extracted from the models generated by depth cameras such as Kinect must take into consideration the natural conditions present on the field. The used sensor should work properly outdoors when the scene has low ambient IR light. The Time-of-Flight distance measurement method is based on measuring the difference between two accumulators in the sensor, each one containing a portion of the returning IR light. However, when sunlight radiation is high the captured radiation on accumulators can be low and this limits the data output. In addition the accuracy depends on the angle of incidence of sunlight. However, several papers have been published evaluating the effect of sunlight in outdoors conditions [42]. Thus the study took constant values of light, time and angle of measurements coinciding with a sunny day at midday. Deformations caused by wind are different from those caused by illumination and they are more difficult to correct by software. In our study, we set up the Kinect Fusion algorithm for a good plant reconstruction at different wind speeds and we extracted values from the created model. The practical relevance of the information generated at high wind speeds is conditioned by the fact that, to avoid the drift of agrochemicals, spraying pesticides or liquid nutrients must be done when wind is almost null. This study confirms the need of considering every weather conditions, including wind speed, when monitoring vegetation outdoors.

## 4. Conclusions

This study has proved that a constant one-direction wind can influence the acquisition of visual depth information. This conclusion is supported by studies conducted with two different tree species (poplars and plum trees) at various wind speeds (up to 10 m·s^−1^). Higher wind speed resulted in higher variability in Kinect v2 height and volume measurement due to tree movement. In general, this sensor was unable to obtain accurate 3D models when wind speed exceeded a 5 m·s^−1^ threshold. Although this threshold was similar for both tree species, individual cases should be studied when performing an outdoor study.

Branch flexibility, leave shape and size affect the fidelity of the 3D models. New algorithms processing maximum height while reducing invalid pixels are required in order to estimate plant volume and height from a single-shot model developed at high wind speeds.
